# Association between shift work and obesity according to body fat percentage in Korean wage workers: data from the fourth and the fifth Korea National Health and Nutrition Examination Survey (KNHANES 2008–2011)

**DOI:** 10.1186/s40557-015-0082-z

**Published:** 2015-12-23

**Authors:** ManKi Son, Byeong Jin Ye, Jung-Il Kim, ShinUk Kang, Kap-Yeol Jung

**Affiliations:** Department of Occupational & Environmental Medicine, College of Medicine, Dong-A University, Busan, South Korea

**Keywords:** Shift work, Obesity, Body fat percentage

## Abstract

**Background:**

Health problems in shift workers vary including obesity acting as a risk factor in cerebrovascular diseases. Recent studies have commonly determined the prevalence of obesity in shift workers on the basis of body mass index. The accuracy of BMI for diagnosing obesity are still limited apparently. Consequently, this study aimed to determine the relationship between shift work and obesity according to the total body fat percentage in Korean wage workers.

**Methods:**

From the Fourth and the Fifth Korea National Health and Nutrition Examination Survey (2008–2011), after military personnel were excluded, a total of 2952 wage workers (20 ≤ age ≤ 65) whose current jobs were their longest jobs were selected as subjects of the study. The total body fat percentage was used to determine the obesity standards (≥25.7 % in males and ≥36.0 % in females). The subjects were divided into groups by gender and work type (manual vs non-manual), and chi-squared test was used to evaluate the relationship between socio-economic, health behavior, and work-related factors, on the one hand, and obesity, on the other. In addition, multivariate logistic regression analysis was performed to examine the effects of shift work on obesity.

**Results:**

When other factors were controlled for, the risk of obesity in shift work showed a statistically significant increase (odds ratio = 1.779, 95 % confidence interval = 1.050-3.015) in the male manual worker group. However, there were no significant results in the male non-manual and female worker groups.

**Conclusion:**

Shift work was related to a higher risk of obesity in the Korean male manual worker group.

## Background

The organization of working time in modern society has diversified with the increasing number of people working in the service sector, the extension of retail hours, and competition and globalization of markets [1]. This diversity includes the form of shift work, a work schedule including irregular or unusual hours of working time. Shift work is reported in > 15 % of the workforce in the EU [1] and U.S. [2]. In the Republic of Korea, the number of working night staff reported in 2011 ranged from 127,000 to 197,000, which constituted 10.2–14.5 % of all wage workers [3].

Health problems that arise in shift workers include obesity, gastrointestinal disorders, cancer, cardiovascular disease, psychological disorders (e.g., depression, insomnia), and work accidents, and shift workers are also affected by the disruption of the circadian system [4–6].

Obesity acts as a risk factor in cerebrovascular diseases such as type 2 diabetes mellitus (DM), cardiovascular disease, hypertension, and cerebral infarction [6]. It is also associated with problems of absenteeism (measured as work loss days), sick leave, disability, injuries, chronic psychological complaints, and emotional exhaustion [7–9].

Recent studies have shown that overweight and obesity are more prevalent in shift workers than day workers. These studies have typically determined the prevalence of obesity in shift workers and day workers on the basis of body mass index (BMI) [10–13]. BMI is the most common method to measure obesity because of its simplicity and low cost. Although a high correlation between BMI and total body fat percentage (TBF%) exists, limitations are still apparent in the accuracy of BMI for diagnosing obesity, particularly for individuals in the intermediate BMI [14]. Furthermore, obesity measured by BMI does not accurately match the degree of adiposity [15–17]. On the other hand, direct assessment of adiposity by TBF% may be a better index of obesity because it correlates better with metabolic and cardiovascular risk factors [15].

Until recently, few studies in Korea have measured obesity using TBF% [18, 19], and no studies have determined the association between shift work and obesity on this basis.

In this study, we aim to measure TBF% using the data of the Korea National Health and Nutrition Examination Survey (KNHANES) and evaluate the risk of obesity in Korean wage workers associated with shift work.

## Methods

### Subjects

This study was based on data acquired in KNHANES IV and V (KNHANES 2008–2011), focusing on the second (2008) and the third year (2009) of the KNHANES IV (2007–2009) survey and the first (2010) and the second year (2011) of the KNHANES V (2010–2012) survey. Data from the year 2008 to the year 2011 have been selected specifically because it is the period when dual-energy X-ray absorptiometry scans were conducted. KNHANES IV and V adopted the rolling sampling survey method in order for three independent rolling samples in each year of the fourth and fifth surveys to be able to represent the nationwide probability sample. Although the sampling frames of the fourth and fifth surveys were different, both have selected their samples using a third level stratified multi-stage clustered probability design. During the process of consolidating the data from the fourth and fifth surveys, when the survey questions were different, they have been integrated.

The total number of subjects of KNHANES from 2008 to 2011 was 31,942. We first included subjects aged above 20 and less than 65 years old. We next included from among them 3,226 wage workers whose current jobs were their longest. Among those, we excluded 264 subjects who were military personnel, pregnant women, reported less than 3 h of sleep per day, or worked less than 10 h per week. Consequently, a total of 2,952 subjects were analyzed.

### Cut-off values (defined as obesity) and body composition analyses

The TBF% was used to set the cut-off value of obesity. Because the cut-off values differ by race-ethnicity [20], cut-off values of Koreans based upon the KNHANES conducted in 2009–2011 were used. These have been determined in a study investigating the optimal cut-offs of the body fat (BF) percentage that may reflect risk factors for cardiovascular disease in Korean adults, with the cut-off values (defined as obesity) set as ≥25.7 % in men and ≥36.0 % in women [19]. The percentage of TBF (total fat mass/total mass × 100) were measured using dual-energy X-ray absorptiometry (DXA; QDR 4500A, Hologic Inc., Waltham, MA, USA) equipment located in the mobile examination centers.

### Classification of data

Independent variables were categorized into socio-economic, health behavior, and work-related factors. Socio-economic factors included age (20–29, 30–39, 40–49, 50–59, 60–65), education level (≤middle school, high school, ≥college), income (first, second quartile, third, and fourth quartiles), marital status (single, married), and menopausal status (postmenopausal, premenopausal). Health behavior factors included alcohol intake (none or social, moderate or heavy), smoking (never smoker, ex-smoker, current smoker), energy intake (<2,000, 2000–2499, ≥2500 kcal), physical activity (light, moderate, vigorous), hours of sleep per day(<7, ≥7), and perceived stress (less, more). Work-related factors included work hours per week (<40, 40–48, 49–60, >60), stability of work (temporary worker, permanent worker), type of job (non-manual, manual), and work schedule (shift work, day work). Energy intake was calculated by a self-administered questionnaire based on the reflection of the past 24 h. The average unit of alcohol consumption was defined as 7 drinks or more for males (5 drinks for females) and subjects were classified as moderate or heavy group, if the respondent consumed more than 2 units per week. Physical activity was measured using the International Physical Activity Questionnaire (IPAQ) [21]. To assess the level of perceived stress in everyday life, subjects were classified into a ‘feel less’ group or ‘feel more’ group by a questionnaire. Jobs were classified by type into manual workers (service and sales workers, agricultural and fishery workers, craft and related trades workers, plant and machine operators and assemblers, and elementary occupations) and non-manual workers (general managers, professionals, paraprofessionals, and office workers). A work schedule between 6:00 and 18:00 was classified as day work, and shift work included a night shift (between 21:00 and 8:00 the next morning), day and night shifts, 24-h work shifts, and irregular shift work. According to the Labor Standards Act (Article 55), night work is defined as working between 10:00 pm and 06:00 am, but in questionnaires of KNHANES, night work was classified as working between 21:00 pm and 8:00 the next morning. So, night shift was classified arbitrarily according to questionnaires of KNHANES.

### Statistical analyses

Because KNHANES is based on a complex survey design, weights, stratified variables, and cluster variables were used for analysis to eliminate any biases. To analyse the combining data of the 4th and 5th KNHANES which are rolling sample survey data, we used the combined weights which was proportional to the size of Primary Sampling Unit.

The general characteristics of the subjects were analyzed by a complex samples chi-squared test for each gender and after classifying non-manual and manual types, a complex samples chi-squared test was again conducted to confirm the association between obesity and independent variables in both genders. Additionally, to determine the risk of obesity resulting from shift work, socio-economic factors, health behaviors, and work-related factors were controlled through complex samples logistic regression. We used SPSS version 18.0 (Chicago, IL, USA) for statistical analysis, and the statistical significance was set at *p* < 0.05.

### Ethics statement

We used data from KNHANES IV and V (KNHANES 2008–2011), including the second and the third year of the KNHANES IV (2007–2009) survey and the first and the second year of the KNHANES V (2010–2012) survey. The data used in this study have previously received consent from the Institutional Review Board of the Korean Centers for Disease Control and Prevention, and the consent numbers are as follows: IRB Nos. 2008-04EXP-01-C, 2009-01CON-03-2C, 2010-02CON-21-C, and 2011-02CON-06-C.

## Results

The general characteristics of subjects with unweighted counts and estimate percentages are shown in Table 1. Among a total of 2952 subjects, there were 1,715 male subjects (66.8 %) and 1,237 female subjects (33.2 %). The percentage of long hours of work exceeding 60 h was higher in male subjects, that is, 11.2 % in the males and 6.4 % in the females. The percentage of temporary workers was 12.0 % and 18.7 % among males and females, respectively, and the percentage of manual workers was 46.8 % and 40.9 %, respectively. The percentage of those performing shift work was 17.7 % and 8.1 % for males and females, respectively, with a higher percentage in the male subjects. The percentage of obesity among the males and females (cut-offs: males: ≥25.7 %, females: ≥36.0) calculated using the total body fat percentage was 26.5 % and 24.2 %, respectively (Table 1).

**Table 1:** General characteristics of the subjects

		Male (*N* = 1715)	Female (*N* = 1237)	Total (*N* = 2952)	*p*-value*
Age group (years)	20-29	69 (1.9)	61 (2.9)	130 (2.2)	<0.001
	30-39	215 (17.5)	334 (35.6)	549 (23.5)	
	40-49	596 (36.2)	318 (22.0)	914 (31.5)	
	50-59	509 (29.5)	326 (25.8)	835 (28.3)	
	60-65	326 (14.9)	198 (13.7)	524 (14.5)	
Education	≤Middle school	210 (10.8)	260 (17.4)	470 (13.0)	<0.001
	High school	579 (36.4)	389 (34.0)	968 (35.6)	
	≥College	926 (52.8)	588 (48.5)	1514 (51.4)	
Income	First quartile	285 (18.3)	244 (19.7)	529 (18.7)	0.388
	Second quartile	431 (26.1)	284 (22.9)	715 (25.0)	
	Third quartile	490 (27.4)	322 (28.1)	812 (27.6)	
	Forth quartile	509 (28.2)	387 (29.3)	896 (28.6)	
Marital status	Single	251 (19.9)	345 (35.3)	2356 (75.0)	<0.001
	Married	1464 (80.1)	892 (64.7)	596 (25.0)	
Alcohol intake	None or social	1288 (75.0)	1177 (94.3)	2465 (81.4)	<0.001
	Moderate or heavy	427 (25.0)	60 (5.7)	487 (18.6)	
Smoking	Never-smoker	355 (21.0)	1123 (89.2)	1478 (43.6)	<0.001
	Ex-smoker	575 (31.0)	62 (6.2)	637 (22.8)	
	Current smoker	785 (47.9)	52 (4.6)	837 (33.6)	
Energy intake (kcal/day)	<2000	461 (25.7)	204 (16.6)	665 (22.7)	<0.001
	2000-2499	543 (32.4)	896 (71.1)	1439 (45.3)	
	≥2500	711 (41.8)	136 (12.2)	847 (32.0)	
Physical activity	Light	599 (34.4)	488 (38.3)	1087 (35.7)	<0.001
	Moderate	541 (31.3)	443 (36.6)	984 (33.0)	
	Vigorous	575 (34.3)	306 (25.1)	881 (31.2)	
Sleep hours per day	<7	735 (43.1)	477 (39.2)	1212 (41.8)	0.072
	≥7	980 (56.9)	760 (60.8)	1740 (58.2)	
Stress	Feel less	1228 (71.5)	807 (64.0)	2035 (69.0)	0.001
	Feel more	487 (28.5)	430 (36.0)	917 (31.0)	
Menopausal status	Premenopausal	-	993 (83.2)	993 (83.2)	
	Postmenopausal	-	244 (16.8)	244 (16.8)	
Work hours per week	<40	139 (7.5)	192 (13.9)	331 (9.6)	<0.001
	40-48	833 (47.7)	702 (57.0)	1535 (50.8)	
	49-60	564 (33.6)	259 (22.7)	823 (30.0)	
	>60	179 (11.2)	84 (6.4)	263 (9.6)	
Stability of work	Temporary worker	198 (12.0)	242 (18.7)	440(14.2)	<0.001
	Permanent worker	1517 (88.0)	995 (81.3)	2512 (85.8)	
Type of job	Non-manual	922 (53.2)	708 (59.1)	1630 (55.2)	0.008
	Manual	793 (46.8)	529 (40.9)	1322 (44.8)	
Work schedule	Shift work	189 (11.7)	86 (8.1)	275 (10.5)	0.009
	Day work	1526 (88.3)	1151 (91.9)	2677 (89.5)	
Obesity	Non-obese	1266 (73.5)	944 (75.8)	2210 (74.3)	0.226
	Obese	449 (26.5)	293 (24.2)	742 (25.7)	

Values are expressed as unweighted count and estimate percentage N(%). *Complex samples χ2 test

Among the male non-manual worker group, the percentage of obesity showed a significant difference depending on physical activity (*p* = 0.044). Among the male manual worker group, the percentage of obesity showed a significant difference depending on income level (*p* = 0.027), stress (*p* = 0.044) and work schedule (*p* = 0.038) (Table 2).

**Table 2:** Characteristics of male subjects by total body fat percentage

		Non-manual		Manual	
		Obese	Non-obese	Obese	Non-obese
		(BF ≥ 25.7)	(BF < 25.7 )	(BF ≥ 25.7	(BF < 25.7 )
Age group	20-29	7 (56.2)	11 (43.8)	10 (23.1)	41 (76.9)
	30-39	23 (23.8)	83 (76.2)	30 (25.3)	79 (74.7)
	40-49	127 (36.3)	235 (63.7)	49 (21.0)	185 (79.0)
	50-59	88 (31.1)	200 (68.9)	39 (17.6)	182 (82.4)
	60-65	40 (27.8)	108 (72.2)	36 (18.4)	142 (81.6)
Education	≤Middle school	3 (22.7)	15 (77.3)	38 (20.5)	154 (79.5)
	High school	48 (28.8)	129 (71.2)	80 (19.3)	322 (80.7)
	≥College	234 (32.9)	493 (67.1)	46 (23.0)	153 (77.0)
Income^b^	First quartile	32 (39.1)	55 (60.9)	36 (19.0)	162 (81.0)
	Second quartile	70 (37.7)	136 (62.3)	58 (27.4)	167 (72.6)
	Third quartile	89 (29.8)	199 (70.2)	42 (18.5)	160 (81.5)
	Forth quartile	94 (27.5)	247 (72.5)	28 (14.4)	140 (85.6)
Marital status	Single	35 (31.1)	83 (68.9)	34 (23.4)	99 (76.6)
	Married	250 (32.0)	554 (68.0)	130 (19.6)	530 (80.4)
Alcohol drinking	none or social	206 (31.2)	479 (68.8)	124 (19.9)	479 (80.1)
	moderate or heavy	79 (33.8)	158 (66.2)	40 (22.0)	150 (78.0)
Smoking	Never smoker	66 (30.1)	154 (69.9)	27 (16.1)	108 (83.9)
	Ex-smoker	85 (28.0)	234 (72.0)	54 (20.9)	202 (79.1)
	Current smoker	134 (35.8)	249 (64.2)	83 (21.7)	319 (78.3)
Energy intake(kcal/day)	<2000	80 (28.7)	188 (71.3)	37 (16.9)	156 (83.1)
	2000-2499	93 (35.5)	199 (64.5)	56 (21.7)	195 (78.3)
	≥2500	112 (31.2)	250 (68.8)	71 (21.4)	278 (78.6)
Physical activity^a^	Light	124 (35.7)	229 (64.3)	60 (24.0)	186 (76.0)
	Moderate	107 (33.2)	226 (66.8)	47 (20.9)	161 (79.1)
	Vigorous	54 (24.7)	182 (75.3)	57 (17.6)	282 (82.4)
Sleep hours per day	<7	135 (32.6)	295 (67.4)	71 (23.5)	234 (76.5)
	≥7	150 (31.2)	342 (68.8)	93 (18.4)	395 (81.6)
Stress†	Feel less	180 (30.3)	425 (69.7)	123 (18.8)	500 (81.2)
	Feel more	105 (34.7)	212 (65.3)	41 (26.3)	129 (73.7)
Work hours per week	<40	21 (46.4)	21 (53.6)	12 (15.2)	85 (84.8)
	40-48	150 (31.0)	341 (69.0)	79 (20.9)	263 (79.1)
	49-60	91 (31.2)	225 (68.8)	45 (18.2)	203 (81.8)
	>60	23 (31.3)	50 (68.7)	28 (28.4)	78 (71.6)
Stability of work	Temporary worker	11 (38.5)	20 (61.5)	31 (20.8)	136 (79.2)
	Permanent worker	274 (31.5)	617 (68.5)	133 (20.4)	493 (79.6)
Work schedule^b^	Shift work	16 (44.0)	24 (56.0)	39 (27.8)	110 (72.2)
	Day work	269 (31.2)	613 (68.8)	125 (18.7)	519 (81.3)

Values are expressed as unweighted count and estimate percentage N(%). ^a^Significantly different between obese and non-obese subjects in non-manual workers (*p* < 0.05). ^b^Significantly different between obese and non-obese subjects in manual workers (*p* < 0.05)

Among the female non-manual worker group, smoking was associated with a significant difference in the prevalence of obesity (*p* = 0.041). Among the female manual workers, menopausal status contributed to significant differences in the prevalence of obesity (*p* = 0.002) (Table 3).

**Table 3:** Characteristics of female subjects by total body fat percentage

		Non-manual		Manual	
		Obese (BF ≥ 36.0)	Non-obese (BF < 36)	Obese (BF ≥ 36.0)	Non-obese (BF < 36.0)
Age group (years)	20-29	0 (0.0)	1 (100.0)	18 (36.6)	42 (63.4)
	30-39	56 (20.5)	221 (79.5)	16 (28.5)	41 (71.5)
	40-49	48 (22.9)	196 (77.1)	15 (22.5)	59 (77.5)
	50-59	24 (16.3)	124 (83.7)	48 (23.1)	130 (76.9)
	60-65	12 (28.7)	26 (71.3)	56 (39.3)	104 (60.7)
Education	≤Middle school	1 (25.0)	4 (75.0)	83 (35.1)	172 (64.9)
	High school	40 (27.9)	128 (72.1)	56 (24.5)	165 (75.5)
	≥College	99 (18.2)	436 (81.8)	14 (27.4)	39 (72.6)
Income	First quartile	16 (19.8)	76 (80.2)	46 (29.0)	106 (71.0)
	Second quartile	36 (28.2)	91 (71.8)	48 (28.8)	109 (71.2)
	Third quartile	36 (22.3)	155 (77.7)	37 (33.1)	94 (66.9)
	Forth quartile	52 (16.0)	246 (84.0)	22 (24.6)	67 (75.4)
Marital status	Single	62 (20.2)	232 (79.8)	16 (28.1)	35 (71.9)
	Married	78 (21.2)	336 (78.8)	137 (29.5)	341 (70.5)
Alcohol intake	None or social	134 (20.4)	548 (79.6)	144 (29.4)	351 (70.6)
	Moderate or heavy	6 (27.1)	20 (72.9)	9 (27.4)	25 (72.6)
Smoking^a^	Never-smoker	124 (19.7)	524 (80.3)	138 (29.5)	337 (70.5)
	Ex-smoker	11 (40.0)	25 (60.0)	8 (29.6)	18 (70.4)
	Current smoker	5 (15.4)	19 (84.6)	7 (26.1)	21 (73.9)
Energy intake(kcal/day)	<2000	24 (19.4)	100 (80.6)	19 (27.8)	62 (72.2)
	2000-2499	95 (20.7)	403 (79.3)	121 (30.3)	277 (69.7)
	≥2500	21 (22.3)	65 (77.7)	13 (24.1)	37 (75.9)
Physical activity	Light	57 (19.5)	249 (80.5)	56 (31.9)	126 (68.1)
	Moderate	63 (23.3)	224 (76.7)	46 (28.2)	110 (71.8)
	Vigorous	20 (17.5)	95 (82.5)	51 (27.8)	140 (72.2)
Sleep hours per day	<7	52 (19.2)	204 (80.8)	64 (29.3)	157 (70.7)
	≥7	88 (21.6)	364 (78.4)	89 (29.2)	219 (70.8)
Stress	Feel less	51 (19.4)	349 (78.5)	101 (26.9)	268 (73.1)
	Feel more	130 (20.6)	219 (80.6)	52 (34.7)	108 (65.3)
Menopausal status^b^	Premenopausal	89 (21.5)	542 (79.4)	78 (24.3)	243 (75.7)
	Postmenopausal	10 (22.9)	26 (77.1)	75 (38.6)	133 (61.4)
Work hours per week	<40	9 (18.8)	56 (81.2)	36 (28.2)	91 (71.8)
	40-48	94 (19.4)	388 (80.6)	66 (30.7)	154 (69.3)
	49-60	35 (26.1)	106 (73.9)	38 (31.5)	80 (68.5)
	>60	2 (11.3)	18 (88.7)	13 (22.3)	51 (77.7)
Stability of work	Temporary worker	12 (23.0)	42 (77.0)	52 (28.0)	136 (72.0)
	Permanent worker	128 (20.5)	526 (79.5)	101 (30.0)	240 (70.0)
Work schedule	Shift work	3 (7.2)	27 (92.8)	14 (29.8)	42 (70.2)
	Day work	137 (21.4)	541 (78.6)	139 (29.2)	334 (70.8)

Values are expressed as unweighted count and estimate percentage N(%). ^a^Significantly different between obese and non-obese subjects in non-manual workers (*p* < 0.05). ^b^Significantly different between obese and non-obese subjects in manual workers (*p* < 0.05)

Table 4 shows the results of the complex samples logistic regression in the male and female subjects. Univariate logistic regression analysis for the male manual worker group showed that the risk of obesity significantly increased with shift work (OR = 1.674, 95 % confidence interval (CI): 1.026-2.733). In multivariable logistic regression analysis controlled for age, education, income, marital status, alcohol intake, smoking, energy intake, physical activity, sleep time, stress, work hours, and stability of work, a higher risk of obesity was significantly associated with shift work (OR = 1.779, 95 % CI: 1.05-3.015). For the female non-manual and manual worker groups, both univariate and multiple logistic regression analyses showed no statistically significant association between shift work and obesity (Table 4).

**Table 4:** Crude and adjusted odds ratio for obesity by selected factors in male and female subjects

	Work schedule	Non-manual workers		Manual workers	
		Crude OR (95 % CI)	Adjusted OR (95 % CI)^a^	Crude OR (95 % CI)	Adjusted OR (95 % CI)^a^
Male	Shift work	1.735 (0.839-3.588)	1.995 (0.948-4.199)	1.674 (1.026-2.733)	1.779 (1.050-3.015)
	Day work	Reference	Reference	Reference	Reference
Female	Shift work	0.285 (0.073-1.111)	0.310 (0.072-1.325)	1.027 (0.483-2.185)	1.325 (0.610-2.877)
	Day work	Reference	Reference	Reference	Reference

Data are shown as odds ratio (95 % confidence interval). ^a^Adjusted for age, education, income, marital status, alcohol intake, smoking, energy intake, physical activity, sleep time, stress, menopausal status(only for female), work hours, stability of work

## Discussion

This study confirmed that obesity measured by total body fat percentage (TBF %) using DXA was associated with shift work among Korean male manual workers. However, there were no association between shift work and obesity according to TBF % in the male non-manual and female manual/non-manual worker groups.

Previous studies that have determined the risk of obesity according to shift work in male manual workers have shown a high prevalence rate of obesity in the shift workers group. In an Italian study of chemical production workers, obesity was more prevalent in shift workers than in day workers [22]. Additionally, similar studies have described an association between shift work and BMI or BMI increase for male manual workers [23, 24]. Upon a year of tracking the BMI of male manual workers in Korea, the three-shift manual workers showed a significant increase in BMI compared to the day workers regardless of working time [23]. In a 14-year cohort study of male workers in a Japanese steel company, alternating shift work had a significant relationship to BMI increase as well [24]. After adjusting the abovementioned covariates in this study, the TBF% of shift workers among male manual workers was significantly higher than that of day workers (*p* = 0.008), as the shift workers’ TBF% was 22.68 % and the day workers’ was 21.11 %, similar to results of previous studies. However, some studies have shown no relationship between shift work and obesity or BMI in male manual workers [25, 26].

Circadian rhythms are endogenously controlled changes in physiology and behavior that enable organisms to regulate their biology in anticipation of predictable daily changes in the environment [27]. Disruption in these rhythms is instrumental in weight gain in shift workers due to the phase shift in the oscillation of the circadian and activity-controlled physiological processes [4]. Circadian disruption may be caused by factors related to inputs, oscillators, and outputs. Inputs includes weak Zeitgebers (caused by continuous light or frequent snacking, for example), conflicting Zeitgebers (resulting from light at night, nocturnal eating, and nocturnal physical activity), and Zeitgeber shifts (daylight-savings time, crossing time zones, shift work). These inputs induce the uncoupling of oscillators and cause the output results of nocturnal melatonin suppression [4]. Supression of melatonin, as during shift work, induces insulin resistance, glucose intolerance, and sleep disturbance leading to obesity [28]. When work schedules disrupt the natural sleep–wake cycle, humans are exposed to light periods when the sun is down, which promote irregular food intake pattern and modifies shift workers’ social and family routines [29]. Irregular food intake pattern such as eating a night time snack affected the thermogenic response that could favor weight gain. Therefore, the time when have a meal may affect the thermogenic response [30]. Recent research has shown evidence for a relationship between altered eating habits, wake–sleep pattern, lifestyle, and obesity; this may explain the increase in obesity among shift workers [31].

Circadian disruption induced by shift work brings loss of cortisol rhythmicity [4]. The hypothalamus controls the secretion of adrenocorticotropic hormone (ACTH) from the anterior pituitary, and ACTH stimulates mainly the secretion of cortisol from the adrenal cortex of glucocorticoid hormones. [32]. Corticotropin-releasing hormone (CRH) plays a principal role in stimulus to the hypo-thalamic-pituitary-adrenal(HPA) axis and a major role as one of the central activators of the integrated stress response [33, 34]. Cortisol is secreted in a circadian rhythm and loss of cortisol rhythmicity could resulting in hyperactivity of the HPA axis, leading to long term elevated cortisol levels [35]. And high levels of cortisol induces obesity [36].

Forced physical exercise often produces negative physiological adaptations to stress responses and can induce the release of CRH. In an animal study, forced wheel running strongly activated CRH neurons compared with spontaneous wheel running, although the amount of exercise was equal [34]. In other studies, plasma ACTH and CRH was increased greater in high intensity exercise than in other group [37], and secretion of ACTH and CRH was related to prolonged physical activity [38]. Prior studies have described an association between long work hours and weight gain in male manual worker [39–41]. Weight gain may be induced by long working through reduced sleep duration, decreased physical activity, and undesirable behaviors [42, 43]. In Korean male manual workers, work hours over 60 h per week increased the risk of obesity [41].

In this study, obesity was associated with shift work among male manual workers, but not in male non-manual workers. Since working over 60 h a week and working condition such as manual work may be acted as a high-intensity physical activity which could cause a physical stress sufficient to increase CRH and ACTH secretion, we presume that association between shift work and obesity was increased in the group.

For female workers, previous studies have reported a significant association between obesity and shift work in manual workers [10, 12] and non-manual workers [13]. In this study, the association between shift work and obesity in female workers has not been consistent in previous studies. There are differences between male and females in regulation of energy metabolism, menstruation, and sex hormones [37, 38], and body fat and weight increases significantly after menopausal in women [44, 45]. In this study, there was a positive relation between obesity and education in male subjects, but a negative relation between the prevalence of obesity in female subjects. The differences of biological gender and socioeconomic factor could account for the gender differences.

There were no association between shift work and obesity in non-manual female workers. Moreover, odds ratios were much lower than 1 although it was not statistically significant. It has shown opposite results compared to previous studies. In this study, among the female non-manual workers, the shift work group had long working hours, although the results did not presented. There were few studies about association between obesity and long working in female non-manual workers, but some studies reported that association between obesity and work hours was not significant in female workers without classifying job type [39, 46]. Chronic work stress may induce suppressed appetite and increased physical activity which lead to weight loss [47], and poor psychosocial working condition may be associated to weight loss [48]. The underlying reasons for the difference in result are not clear. Shift work involved long working may be viewed as a chronic work stress that was sufficient to induce weight loss in female non-manual workers, although we did not evaluate work stress or psychosocial working condition. In addition, prior studies have investigated a single job class; however, they have not been designed to evaluate the general characteristics of national female manual and non-manual workers.

Because this is a cross-sectional study based on survey data, there may be bias according to the subject’s memory of the past. Furthermore, although we have selected our subjects to include workers whose current jobs are their longest jobs to identify a stronger relationship with work schedule, the present study could not determine working period for studying the effect of shift work. Data of the energy intake was only evaluated by a questionnaire based on reflection self report of the past 24 h. Despite these limitations, this study is meaningful because the data was based on survey representing the Korean general population and this study identified association between shift work and obesity measured by TBF% according to gender and job type (manual vs. non-manual work). In addition, not only were age, socio-economic factors, health behavior, and work-related factors adjusted for, but also adiposity was directly calculated by using total body fat percentage, setting the standard for defining obesity and thereby the evaluation of the association between shift work and obesity. Shift work was related to a higher risk of obesity according to body fat percentage in the Korean male manual worker group. The male manual workers engaging in shift work should be considered as a high risk group for obesity, and workplace weight management program should be focused on the group. Further studies should need to consider subjects’ dietary habits such as usual volume of consumption, eating pattern, and, nutrition quality, and working period, level of occupational physical stress, and job stress.

## Conclusion

In this study based on survey representing the Korean general population, we confirmed that obesity measured by total body fat percentage (TBF %) using DXA was associated with shift work among Korean male manual workers. However, there were no association between shift work and obesity in the male non-manual, female manual and non-manual worker groups. Consequently, the male manual workers engaging in shift work should be considered as a high risk group forobesity.
